# Gene Expression Profiling of Human Oocytes Developed and Matured *In Vivo* or *In Vitro*

**DOI:** 10.1155/2013/879489

**Published:** 2013-02-20

**Authors:** Irma Virant-Klun, Katja Knez, Tomaz Tomazevic, Thomas Skutella

**Affiliations:** ^1^Reproductive Unit, Department of Obstetrics and Gynecology, University Medical Centre Ljubljana, Slajmerjeva 3, 1000 Ljubljana, Slovenia; ^2^Institute for Anatomy and Cell Biology, Medical Faculty, University of Heidelberg, 69120 Heidelberg, Germany

## Abstract

The quality of the human oocyte determines the success of fertilization and affects the consequent embryo development, pregnancy and birth; it therefore serves as a basis for human reproduction and fertility. The possibility to evaluate oocyte quality in the *in vitro* fertilization programme is very limited. The only criterion which is commonly used to evaluate oocyte quality is its morphology. There is a mass of oocytes in the *in vitro* fertilization programme which are not fertilized in spite of normal morphology. In the past, several attempts focused on oocyte gene expression profiling by different approaches. The results elucidated groups of genes related to the human oocyte. It was confirmed that some factors, such as oocyte *in vitro* maturation, are detectable at the molecular level of human oocytes and their polar bodies in terms of gene expression profile. Furthermore, the first genetic evaluations of oocyte-like cells developed *in vitro* from human stem cells of different origin were performed showing that these cells express some genes related to oocytes. All these findings provide some new knowledge and clearer insights into oocyte quality and oogenesis that might be introduced into clinical practice in the future.

## 1. Introduction

A viable quality human oocyte is a prerequisite for successful fertilization, implantation, and development of a new human being. Although an enormous development of techniques of *in vitro* fertilization has arisen in recent years and several indications of infertility became treatable, the general implantation and pregnancy rates after the embryo transfer are still low [1]. A mass of embryos are developed in the *in vitro* fertilization programme which do not implant. This is mainly due to a lack of reliable criteria on oocyte quality and the selection of embryos with sufficient developmental potential. The quality of oocytes obtained under ovarian stimulation for *in vitro* fertilization varies considerably and depends on several factors. A large proportion of oocytes are capable of being fertilized, but only approximately half of fertilized oocytes fully complete their preimplantation development and still fewer implant [2]. More studies have evidenced that some gene expression levels could serve as oocyte quality markers. Additionally, recent studies also confirmed that oocyte-like cells expressing some oocyte-specific genes can be developed *in vitro* from different types of stem cells. These include human embryonic stem cells (hESCs) [3–7], human induced pluripotent stem cells (hiPSCs) [6, 7], putative stem cells from adult human ovaries of women with no naturally present follicles or oocytes [8–10], and human amniotic fluid stem cells (hAFSCs) [11]. Therefore it is also important to know the gene expression profile of these cells to evaluate their quality and safety. The aim of this paper is to summarize the new knowledge on the gene expression profile of human oocytes developed and matured *in vivo* and *in vitro*. 

## 2. Human Oocyte

An oocyte is a female germ cell involved in reproduction. It is one of the largest cells in the body with a diameter of at least 100 *μ*m when mature. The oocyte is rich in cytoplasm that contains yolk granules to support the cell's growth, maturation, and the early development after fertilization. *In vivo* it is produced in the ovarian follicle, a functional unit of the ovary, during the process of oogenesis/folliculogenesis [12]. The oogenesis then starts with the development of oogonia. Each oogonium inside the fetal ovaries divides and enters the initial stage of meiosis (meiosis I) to become the *primary oocyte*. The diploid *primary oocyte,* however, does not complete meiosis I but is stopped at the first meiotic prophase stage, called dictyate. At this stage the oocyte nucleus is called the *germinal vesicle* (GV), as this stage refers to the GV stage of maturity. GV oocytes are localized within the primordial follicle, also consisting of a flattened and condensed layer of surrounding mesenchymal granulosa cells. By the end of the fetal period, all *primary oocytes* have formed and are stopped at the dictyate stage. Although meiosis was arrested, the dictyate chromosomes continue to synthesize large amounts of mRNA and rRNA, which are later used to generate a bulk of essential proteins needed for oocyte maturation and further development of the fertilized oocyte and embryo [12]. Then the *primary oocytes* are maintained for years, until puberty (menarche). Upon puberty the *primary oocytes* finish meiosis I, and a *primary oocyte* divides into two daughter cells: a haploid *secondary oocyte* and an extruded nonfunctional polar body. *During* the menstrual cycle only a few of *primary oocytes* are recruited, and only one matures and is ovulated. When this secondary oocyte enters meiosis (meiosis II), it does not finish but is arrested again and held at the metaphase II (MII) stage until fertilization. The MII-stage oocyte has the potential to be fertilized. When the oocyte is fertilized, the process of meiosis is terminated and the second polar body is extruded. During the menstrual cycle only 15–20 early antral follicles/oocytes are recruited and only one dominant follicle matures fully and is ovulated. Each oocyte develops and matures in the functional unit of the ovary—the follicle. The *primary oocytes* grow and mature during the development and maturation of primordial, primary and secondary follicles, whereas the secondary oocytes develop along with the tertiary and preovulating Graafian follicle. This process is regulated by hormones and other substances.

**Figure 1:**
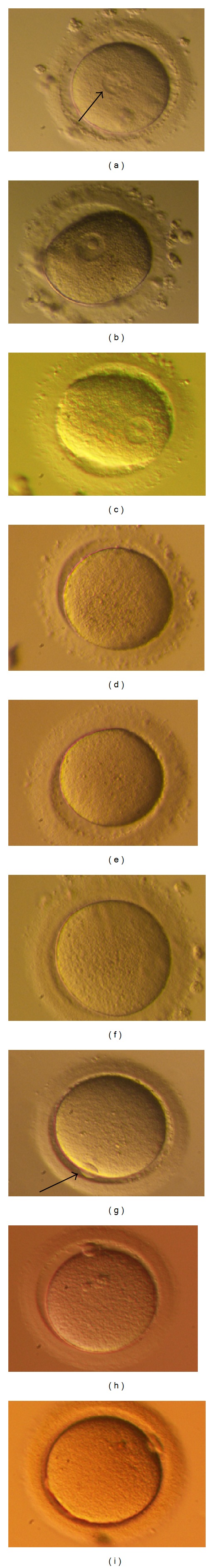
Human oocytes from the *in vitro* fertilization programme (a)–(c): immature germinal vesicle (GV) oocytes with a germinal vesicle (arrow) and without a polar body; (d)–(f): immature metaphase I (MI) oocytes without a polar body; (g)–(i): mature metaphase II (MII) oocytes with a polar body (arrow).

In the *in vitro* fertilization programme oocytes are retrieved after hormonal stimulation of patient's ovaries and by ultrasound guided aspiration. Oocytes at different stages of maturity can be retrieved. Most oocytes are mature metaphase II (MII) oocytes, while a relatively small proportion of oocytes are immature metaphase I (MI) oocytes and prophase I (PI) or germinal-vesicle- (GV-) stage oocytes. The MII oocytes are characterized by a round shape, zona pellucida, and an extruded polar body; MI oocytes by a round shape, zona pellucida, and the absence of a polar body; GV-stage oocytes by a round shape, zona pellucida, and the presence of a germinal vesicle (for morphology see Figure 1). In the *in vitro* fertilization programme only mature MII-stage oocytes can be fertilized and develop into an embryo; therefore, the immature oocytes need to be matured *in vitro* or discarded in daily clinical practice. 

Because the main role of an oocyte is to be fertilized and to grow into a fully functional organism, it has to be able to regulate many different cellular and developmental processes, such as the regulation of the cell cycle progression and cellular metabolism, fertilization, activation of zygotic transcription, embryo development, activation of the embryonic genome, and formation of body axes. During oocyte growth a variety of maternally transcribed mRNAs are supplied which represent the maternal contribution to the oocyte and, consequently, the newly fertilized oocyte, zygote, and early embryo. These mRNAs can be stored in message ribonucleoprotein (mRNP) complexes and then translated when needed [13]; additionally, they can be localized within a specific region of the cytoplasm or dispersed within the cytoplasm of the entire oocyte. Maternally synthesized proteins can also be localized or ubiquitous throughout all the oocyte's cytoplasm. 

## 3. Gene Expression Analyses of Human Oocytes 

The first gene expression analyses in human oocytes were performed by reverse transcription-polymerase chain reaction (RT-PCR; see Table 1) and were followed using microarray technology (Table 2). Microarrays enable gene expression profiling, which gives the researcher the ability to monitor and quantify the expression of thousands of known genes at the same time (e.g., whole genome microarray). This methodological breakthrough has the potential to provide detailed insight into cellular processes involved in the regulation of gene expression in different cell types, including oocytes and cumulus cells.

### 3.1. Human Oocyte-Specific Genes

More studies elucidated the oocyte gene expression profile from different aspects; most of these studies used the microarray technology and are listed in Table 2. In the study by Bermúdez et al. [14], microarray methods were used to examine the expression of linearly amplified RNA from individual oocytes and groups of five oocytes. In this study the amplification strategy consistently enabled a complex representative cDNA population. With this methodology a catalogue of 1,361 different transcripts expressed in human oocytes was identified; 406 of them have been independently confirmed by other methods. According to their function, the expression of several genes was related to apoptosis, cell cycle, circadian rhythms, cytoskeleton, secretory pathways, exocytosis, endocytosis, kinases, membrane receptors, ion channels, mitochondria, structural nuclear proteins, phospholipases, protein degradation, protein synthesis, secreted proteins, signaling pathways, DNA, chromatin, RNA, transcription, and others. The results of this study highlighted the potential of microarray analyses in reproductive medicine. 

Using microarray analysis, Kocabas et al. analyzed the transcriptome of non-fertilized mature MII oocytes from the *in vitro* fertilization programme [15]. They analyzed three groups of 10 oocytes from three young donors, each less than 35 years of age, reproductively healthy with regular ovulatory cycles, with the male factor as the only cause of infertility, and a considerable number of developing follicles. The transcriptome of these oocytes was compared to 10 different normal human tissues, including skeletal muscle, kidney, lung, colon, liver, spleen, breast, brain, heart, and stomach. Compared with reference samples, there were 5,331 transcripts significantly up-regulated and 7,074 transcripts significantly down-regulated in the human oocytes. Genes up-regulated in oocyte samples included most of the well-known germ cell-specific genes. The authors confirmed the presence of pathways previously described in the mouse oocytes, especially the TGF-*β*. Among genes up-regulated in oocytes, 1,430 were of unknown function. A group of 66 genes up-regulated in human oocytes was identified by intersecting significantly up-regulated genes in human oocytes with those from the mouse oocytes and from human and mouse embryonic stem cells. Additionally, microarray results were validated using RT-PCR analysis for a selected set of oocyte-specific genes. The genes up-regulated in human oocytes were related to RNA, DNA, protein metabolism, and chromatin modification. The overexpression of genes associated with RNA metabolism is in agreement with the fact that oocytes store a great amount of RNA to support the processes of fertilization, early embryonic development, and activation of the embryonic genome. The up-regulation of genes related to DNA metabolism, transcription regulation (zinc finger proteins), and chromatin modification is in agreement with the oocyte need to remodel the sperm chromatin after fertilization. The human oocyte is proposed to be transcriptionally silent at the MII stage of maturity but is very active in transcription and translation during the growth phase and must be prepared to initiate transcription during embryonic genome activation at the 4- to 8-cell embryo stage. The authors concluded that further understanding of the biological role of genes up-regulated in mature human oocytes may extend the knowledge on the meiotic cell cycle, fertilization, chromatin remodeling, lineage commitment, pluripotency, tissue regeneration, and morphogenesis. Moreover, Assou et al. found 1,514 genes which were up-regulated in human oocytes in addition to already known genes, such as *DAZL*, *BMP15,* or *GDF9*. Among them were the meiosis-related genes *PTTG3* (securing) and *AURKC* (Aurora kinase), previously unreported growth factors such as *TNFSF13/APRIL*, *FGF9*, *FGF14* and *IL4*, as well as transcription factors, including *OTX2*, *SOX15, *and *SOX30 *[16]. The most specific and significantly up-regulated oocyte genes, summarized from the perviously mentioned manuscripts, are listed in Table 3. In spite of some complex gene expression profilings of human oocytes, the relation of expressed genes to the fertilization process, embryo development, and conceiving is still poorly understood and needs to be elucidated in the future.

Further attempts were made to optimize a microarray-based approach for deriving representative gene expression profiles of human oocytes. Since the human oocyte is a low RNA template sample which contains approximately 55 to 100 pg of polyA+ mRNA [20, 21], it was proposed that an amplification step is required to provide sufficient labeled RNA as a microarray target. Jones et al. tried to optimize a protocol for deriving reproducible and representative gene expression profiles by microarrays from very rare samples of human oocytes, available for research purposes [22]. In their study cRNA was generated from both single and pooled immature human GV-stage oocytes. The amplification products were used as a microarray target to optimize a protocol of gene expression profiling. They found that linear amplification and exponential amplification were both capable of generating sufficient products for hybridization to the microarrays, even from the low amount of template mRNA present in a single human oocyte. The results of this study showed that the majority of the variance associated with amplification and hybridization procedures resulted from the molecular processing; therefore, oocytes need to be pooled for the starting template for each array and sufficient independent microarray experiments need to be performed to minimize the variance associated with molecular processing. In this study the hierarchical cluster analysis of the five immature GV-stage oocytes and the two MII-stage oocytes showed significant deviation in branch distances despite the fact that the oocytes were retrieved from women of similar age and indication of infertility.

**Figure 2:**
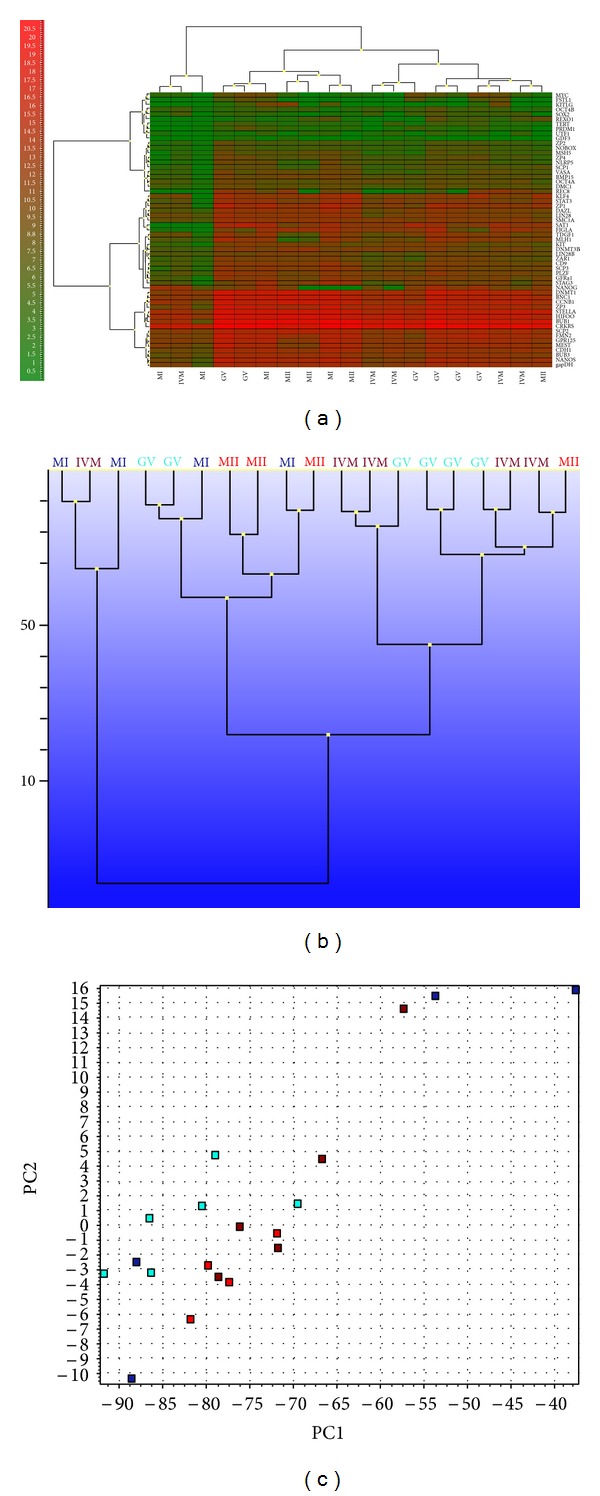
Analyses of nineteen human oocytes at different stages of maturity on the expression of fifty-six genes related to pluripotent stem cells and oocytes using a Fluidigm Real-Time system confirmed three outstanding oocytes (two MI and one IVM oocyte). (a) heatmap clustering (Ward's Algorithm, Euclidean Distance Measure), (b) hierarchical clustering (Ward's Algorithm, Euclidean Distance Measure), (c) principal component analysis (PCA) *legend for *(a) red—expressed, green—nonexpressed; *legend for *(b)* and *(c) aquamarine—GV oocytes, blue—metaphase I oocytes, red—MII oocytes, dark red—IVM oocytes. Analyzed genes: *VASA*, *GPR125*, *DAZL*, *KIT*, *KIT-LIG*, *STELLA*, *GFRa1*, *PLZF*, *OCT4B*, *OCT4A*, *LIN28*, *GDF3*, *NANOG*, *MYC*, *KLF4*, *SOX2*, *UTF1*, *TDGF1*, *DNMT3B*, *LIN28B*, *TERT*, *CD9*, *NANOS*, *CDH1*, *STAT3*, *REX01*, *DNMT1*, *BMP15*, *ZP1*, *ZP2*, *ZP3*, *ZP4*, *SCP1*, *SCP2*, *SCP3*, *SMC1A*, *FSTL1*, *CCNB1*, *BNC1*, *BUB1*, *BUB3*, *NOBOX*, *MSH5*, *NLRP5*, *FMN2*, *HIFOO*, *MEST*, *CRKRS*, *MLH1*, *ZAR1*, *REC8*, *PRDM1*, *SAT1*, *FIGLA*, *STAG3*, and *DMC1. *

Our own preliminary data show that single human oocytes from the *in vitro* fertilization programme can also be successfully analyzed by the Biomark Real-Time Quantitative PCR (qPCR) System (Fluidigm). This methodology enables qualitative analyses of single cells after preamplification. The advantage of the methodology is that many genes can be analyzed at the same time. In our preliminary work 19 single human oocytes at different stages of maturity (6 GV, 4 MI, 5 *in vitro* matured (IVM), and 4 mature MII oocytes) were analyzed on 56 genes related to pluripotent stem cells and oocytes by the Fluidigm system after the preamplification procedure. The analyzed genes were selected to elucidate the relation of human oocytes to hESCs and to better understand the molecular status of oocyte-like cells developed *in vitro* from stem cells of different origins, including pluripotent hESCs and iPSCs. The inventoried TaqMan assays (Applied Biosystem) were pooled to a final concentration of 0.2× for each assay. The cells to be analyzed were harvested directly into RT-PreAmp Master Mix. The harvested cells were immediately frozen and stored at −80°C. Cell lysis and sequence-specific reverse transcription were performed at 50°C for 15 minutes. The reverse transcriptase was inactivated by heating to 95°C for 2 minutes. Subsequently, cDNA went through limited sequence-specific amplification by denaturing at 95°C for 15 seconds and annealing and amplification at 60°C for four minutes for 14 cycles. These pre-amplified products were diluted 5-fold prior to analysis with the Universal PCR Master Mix and inventoried with TaqMan gene expression assays in 96.96 Dynamic Arrays on a BioMark System. Ct values obtained from the BioMark System were transferred to the GenEx software (MultiD) to analyze the gene expressions. The results confirmed that a small proportion—three outstanding oocytes (two immature MI oocytes and one *in vitro* matured oocyte IVM)—differed at the molecular level from other oocytes and did not cluster with them, as revealed by heatmap, hierarchical clusterings, and principal component analysis (Figure 2). Although no statistical differences in gene expression were found between them, we concluded that a higher number of single oocytes at each stage of maturity would have to be analyzed using this methodology in the future. 

### 3.2. Genes Related to Preimplantation Development

Still little is known about gene expression in human oocytes and during early embryo development because of the rare availability of materials to be researched however some studies have tried to elucidate this (see Tables 1 and 2). 

Wells et al. quantified the expression of nine different genes *BRCA1*, *BRCA2*, *ATM*, *TP53*, *RB1*, *MAD2*, *BUB1*, *APC,* and *beta-actin* in four mature human oocytes and forty-two embryos by RT-PCR [17], as can be seen in Table 1. These genes are known to play key roles in processes important for preimplantation development, such as cell cycle regulation, DNA repair, signaling pathways, construction of the cytoskeleton, and apoptosis. The authors found that the analyzed oocytes showed relatively high levels of mRNA transcripts, while 2-3-cell preimplantation embryos were found to contain very little mRNA from any of the genes analyzed. The recovery of gene expression levels was not seen until the 4-cell stage embryo or later, which may be related to the activation of the embryonic genome. During preimplantation development of embryos some genes displayed great increases in expression at the stage of 4–8 cells, but for most genes the maximal expression was not achieved until the blastocyst stage. From these results it may be concluded that it is possible to define characteristic gene expression profiles for each stage of human preimplantation development. The identification of genes expressed at different preimplantation phases of development may provide the link to the cellular pathways which are activated at defined stages of oocyte development. The expression of genes related to DNA repair pathways in cleavage stage embryos indicated that DNA damage may be a common feature at this stage. It was suggested that specific profiles of gene expression may be indicative of embryo development and implantation potential. In another study Li et al. analyzed the gene expression of human lactate dehydrogenase isozymes (*LDH-A*, *LDH-B*, and *LDH-C*) and small ubiquitin-like modifier isoforms (*SUMO-1*, *SUMO-2*, and *SUMO-3*) in four single MII-stage oocytes, two 4-cell and three 8-cell embryos using the reverse transcription-polymerase chain reaction [18]. The mRNAs for SUMO-1, SUMO-2, SUMO-3, and LDH-B (heart) were detected in all oocytes and 4- and 8-cell embryos. The mRNA for LDH-A (muscle) was detected in two of four oocytes and in one of three 8-cell embryos. Lactate and pyruvate are the crucial nutrients for the cleavage stage embryos of many mammalian species, including humans, as confirmed also by this study. The interconversions of lactate and pyruvate are catalyzed by lactate dehydrogenase (LDH), which was expressed in oocytes and embryos in this study. Small ubiquitin-like modifier (SUMO) proteins in humans, expressed in oocytes and embryos in this study, are involved in protein trafficking and targeting during posttranslational modification. The mRNA for testis-specific LDH-C was not detected in any sample in this study.

Using microarray technology Dobson et al. analyzed gene expression in single human oocytes and preimplantation embryos to elucidate the gene expression profile during human preimplantation development from the oocyte to the 8-cell stage embryos [21]. The results of this study provided the first global analysis of the human preimplantation embryo transcriptome and demonstrated that RNA can be successfully amplified from single oocytes and embryos for analysis by cDNA microarray technology. To identify the genes whose transcript levels changed throughout the first 3 days of preimplantation development, they analyzed gene expression in single oocytes (five primary oocytes and two secondary oocytes) and embryos (seven day 1 embryos, three day 2 embryos, and five day 3 embryos), each compared to a control primary oocyte. They found that a specific pattern of gene expression exists with most genes that are transcriptionally modulated during the first three days following fertilization being not up-regulated but down-regulated in comparison with oocytes. They also observed that the majority of genes which showed differential expression during preimplantation development were of unknown identity and function and that embryonic transcriptional programs were clearly established by day 3 following fertilization, even in embryos that arrested prematurely at 2-, 3- or 4-cell stages. For the first time it was indicated that the failure to activate transcription is not associated with the majority of human preimplantation embryo loss in the *in vitro* fertilization programme. In the study of Li et al. the cDNA microarray from single oocytes and 4- and 8-cell embryos were used to elucidate the differential expression profiles [18]. In oocytes 184 genes were found to be expressed more than twofold above the median value, but only two genes were at least twofold below the median value. In 4-cell embryos 29 genes were expressed more than twofold above the median value, but 98 genes expressed at least twofold below the median value. In 8-cell embryos 65 genes were found to have a value more than twofold above the median value, and 287 genes were expressed at least twofold below the median value of all genes expressed in oocytes and embryos. This indicated that the expression of some zygotic genes had already occurred in 4-cell embryos.

### 3.3. Gene Expression Profile during Oogenesis/Folliculogenesis

Huntriss et al. studied the expression of the *NOBOX* gene in human ovarian follicles and oocytes and presented the first cDNA cloning and transcript expression analysis of the human *NOBOX* gene using RT-PCR [19], as can be seen in Table 1.* NOBOX* is a homeobox gene that is preferentially expressed in the oocytes and ovarian follicles and was first identified in the mouse. When analyzing adult human tissues, they found that the expression of this gene is limited to the ovary, testis, and pancreas. The *NOBOX* gene expression within the ovary was oocyte-specific and was found from the primordial stage of ovarian follicles through to the mature MII oocytes. Additionally, Huntriss et al. analyzed the expression profiles of 14 additional homeobox genes during human oogenesis and early development. Using RT-PCR they found that the expression of *HOXA10* was limited to primordial and early primary follicles. The gene *HOXB7* was expressed from primordial and early primary stage follicles to the GV-stage oocytes. The homeobox genes gastrulation brain homeobox 1 (*GBX1*) and *HOXA7* were predominately expressed by GV oocytes and *HOXA1* and *HEX* by MII oocytes. It was concluded that the homeobox gene transcripts detected in ovarian follicles and oocytes were distinct from those expressed in human embryos at the blastocyst stage (*HOXB4*, *CDX2,* and *HOXC9*) and in granulosa cells (*HOXC9*, *HOXC8*, *HOXC6*, *HOXA7*, *HOXA5,* and *HOXA4*). 

Markholt et al. have isolated pure populations of oocytes from the early follicles at different stages of maturity—primordial, intermediate, and primary follicles—by laser capture microdissection methodology and analyzed them by whole-genome microarray analysis [23]. The microarray data were also confirmed by qPCR for selected genes. Their results confirmed that a total of 6,301 unique genes were significantly expressed representing categories of genes related to “RNA binding,” “translation initiation,” and “structural molecule activity”. Several genes were also found to be associated with early oocyte development, where some were identified with extraordinarily high expression levels, such as the antiproliferative transmembrane protein with an epidermal growth factor-like and two follistatin-like domains (*TMEFF2*): the Rho-GTPase-activating protein oligophrenin 1 (*OPHN1*) and the mitochondrial-encoded ATPase6 (*ATP6*). The genes *TMEFF2 *(also known as *Tomoregulin-2*) and *OPHN1* were among the most expressed genes overall. In oocytes from primordial/primary follicles the mitochondria-related gene *DC48 *was also strongly expressed. In the oocytes from the primordial/primary follicles the genes related to ovarian steroidogenesis were not expressed or were only weakly expressed. In terms of steroid receptors the androgen receptor (*AR*), estrogen receptor 2 (*ER2*), and the progesterone receptor membrane component 1 + 2 were expressed at a low level, while there was no expression of the follicle-stimulating hormone receptor (*FSHR*) and luteinizing hormone receptor (*LHR)*. Among transcription factors several genes from the POU and Forkhead families were expressed in oocytes from early follicles: *POUF3F2* (*OCT-3*), *POU4F2*, *POU2F1* (*OCT-1*), *POU5F1 *(*OCT-4*), and *FOXO1*, *FOXO3*, *FOXR1*, *FOXP1*. Among TGF*β* family of growth factors and other growth factors were several genes which were not expressed: bone morphogenetic protein (*BMP15*), anti-Mullerian hormone (*AMH*), *AMH* receptor 2 (*AMRH2*), activin/inhibin subunits, insulin-like growth factor I (*IGFI*), platelet-derived growth factor (*PDGF*) and its receptor, and epidermal growth factor (*EGF*) and its receptor (EGFR) were not expressed in appreciable levels, while in contrast a number of BMP receptors (*BMP1A* and *BMP1B*), activin receptors (*activin 1B*, *2A*, and *2B*), *IGFI* receptor, fibroblast-growth factor (*FGF*)-*9*, *FGF14*, *FGF* receptor 4, and transforming growth factor alpha (*TGF*α**) were consistently expressed. In oocytes from early follicles several genes of signal transduction were expressed, such as genes for enzymes phosphatidylinositol 3-kinase (*PI3K*), *PI4K*, and *PTEN* that regulate the levels of phosphatidylinositol 3,4,5-triphosphate and genes for phosphodiesterases (*PDE4D*, *PDE8A + B,* and *PDEI2*) and for the mitogen-activated protein kinase I (*MAPKI*). In oocytes from primordial follicles some oocyte or germline-specific genes were found to be expressed, such as the factor in the germline alpha (*FIGLA*), zona pellucida glycoproteins *ZP1*, *ZP2*, *ZP3*, *ZP4*, spermatogenesis and oogenesis-specific basic helix-loop-helix I (*SOLHI*), *SOLH2*, the newborn ovary homeobox (*NOBOX*) gene, the maternal embryonic leucine zipper kinase (*MELK*), and oocyte-specific gene (*MATER*). The authors proposed a technique of laser capture microdissection combined with transcriptome analysis of the rare material of human oocytes from the early follicle stages as an important tool to gain better understanding of early human folliculogenesis and oogenesis. These results indicated some genes which might be related to the oocyte maturation process.

### 3.4. Gene Expression Profiles in Human Oocytes and Human Embryonic Stem Cells

The real origin of hESCs is still a matter of debate. Thus far it has been believed that they most closely resemble pluripotent primitive ectoderm cells derived from the blastocyst inner cell mass. However, differences between ESCs and primitive ectoderm cells have opened the question whether ESCs really have an *in vivo* equivalent or whether their properties mostly reflect their artificial culture environment. Some authors have proposed that they might be related to the germinal lineage [35]. Early human preimplantation embryo development is characterized by the induction of totipotency, followed by pluripotency. The understanding of this very complex process could be implicated among *in vitro* fertilization methodology and regenerative medicine. Human mature MII-stage oocytes and hESCs have some common features: both are able to achieve cell reprogramming towards pluripotency in different ways (e.g., by somatic cell nuclear transfer and cell fusion). The comparison of the transcriptome of these two types of cells may highlight some genes that are involved in the pluripotency initiation. Zhang et al. confirmed the expression of distinct sets of developmentally regulated genes that are expressed by both the human oocytes and hESCs [24]. The main point of their study was to identify genes that were expressed differently during final oocyte maturation and early embryonic development in humans. Using the microarrays and RT-PCR methodology they compared gene expression profiles of 76 human GV-stage oocytes from 55 donor patients included in the *in vitro* fertilization programme, hESCs, and human foreskin fibroblasts. In their study 10,183 genes were expressed in human GV oocytes, and 45% of these genes were still unclassified by their biological function. Four oocyte-specific genes—*MATER*, *ZAR1*, *NPM2,* and *FIGLA*—were for the first time detected in human GV-stage oocytes. Additionally, the components of 4 signaling pathways—MOS-MPF, TGF-*β*, Wnt, and Notch—were also found to be expressed in human GV oocytes. Distinct sets of genes were detected by comparison of gene expression profiles between human GV oocytes, hESCs, and human foreskin fibroblasts, and these gene sets may be involved in the processes of oocyte maturation and early embryonic development. Furthermore, Assou et al. compared the gene expression profile of mature MII oocytes from the *in vitro* fertilization programme and hESCs to that of somatic tissues [25]. They identified a common oocyte/hESC gene expression profile, which included several genes involved in the cell cycle, such as those encoding enzymes involved in general cell metabolism (*METAP2*, *SHMT2*, etc.), nucleoside synthesis (*DHFR*, *TYMS*, *RRM2*, *PPAT*, etc.), DNA repair including mismatch repair (*MSH2 *and *MSH6*) or base excision repair (*UNG *and *PCNA*), main components of the cell cycle regulatory machinery (*CCNB1 *and *2*, *CCNA*, *CCNE*, etc.), regulators of the topologic state of DNA (*TOP1* and *TOP2A*) and components of the mitotic spindle assembly checkpoint (the centromere constituents *CENPE*, the securin *PTTG1*, and *MAD2L1*, *BUB1B*, *BUB3*), genes related to pluripotency (e.g., *LIN28 *and *TDGF1)*, large chromatin remodeling network genes (e.g., *TOP2A, DNMT3B, JARID2, SMARCA5, CBX1, and CBX5*), 18 different zinc finger transcription factors, including *ZNF84*, and several genes which are still poorly understood, such as *KLHL7*, *MRS2*, or the selenophosphate synthetase 1 (*SEPHS1*). Additionally, a large set of genes was also found to encode proteins involved in the ubiquitination and proteasome pathways. After differentiation of hESCs into embryoid bodies, the transcription of these gene pathways was found to decline. These data indicated the important relation of oocytes to hESCs but also some important oocyte-specific differences. 

### 3.5. Oocyte-Like Cells Developed *In Vitro*

There were also several successful attempts to culture oocyte-like cells (OLCs) from hESCs [3–7], hiPSCs [6, 7], hOSCs [8–10], and hAFSCs [11]. These OLCs expressed some oocyte-specific genes, as revealed by RT-PCR. The data are summarized in Table 4. West et al. found that the enrichment and differentiation of human germ-like cells were mediated by mouse embryonic fibroblast (MEF) feeder cells and basic fibroblast growth factor (bFGF) [3]; these OLCs significantly up-regulated genes related to premigratory/migratory processes, as well as some meiosis-related genes, as can be seen in Table 4. Furthermore, they cultured OLCs from hESCs and confirmed that endogenous BMP expression caused germ-like (DDX4 (VASA)+ POU5F1+) cell differentiation, and the inhibition of this pathway resulted in a significant decrease of germ cell-related gene expression and the number of germ-like cells [4], as also presented in Table 4. Additionally, they confirmed that the loss of KITL in the culture system resulted in a significant down-regulation of germ cell genes and a 70.5% decrease of germ-like cells. Their results indicated that both the BMP and KITL expressions are important for *in vitro* development of hESC-derived germ-like cells and that they may play an important role in human oogenesis. 

Richards et al. [5] cultured embryoid bodies from hESCs in six different culture conditions (mitotically inactivated porcine ovarian fibroblasts, 100% conditioned medium from porcine ovarian fibroblasts, 50% conditioned medium from porcine ovarian fibroblasts, forskolin, transretinoic acid RA, and forskolin and RA) and in all cultured conditions confirmed the development of OLCs, which expressed several oocyte-specific genes, such as *VASA*, *DAZL*, *GDF3*, *GDF9*, *MLH1*, *SCP1*, *PUM1*, *PUM2,* and *POU5F1 *(see Table 4). Among all culture conditions, porcine ovarian fibroblasts were proven to be the best system for initiating germ cell differentiation *in vitro*. 

Medrano et al. [6] found that with overexpression of *VASA *and/or *DAZL *genes, both the hESCs and iPSCs were differentiated into primordial germ cells, and maturation and progression through meiosis was enhanced. These cells expressed several genes related to early germ cells (*VASA*, *DAZL*, *FITM1*, *PRDM1A*, *GCNF*, *GDF3*, *CKIT*, *PELOTA*) and late germ cells and meiosis (*SCP3*, *MLH1*, *DMC1*, *GDF9*, *ZP4*), as can be seen in Table 4. Moreover, Eguizabal et al. [7] have recently reported that hESCs and hiPSCs were successfully differentiated into OLCs and then successfully passed through the process of meiosis, were haploid, and expressed several genes related to early and late germ cells as well as the process of meiosis (Table 4). This was the first report indicating that OLCs developed *in vitro* from stem cells might pass through the process of meiosis and finally differentiated in haploid cells. 

The OLCs and parthenogenetic-like structures were developed from the putative human ovarian stem cells from the ovarian surface epithelium of women with no naturally present oocytes and follicles—postmenopausal women and women with premature ovarian failure—as reported by our group [8–10]. These *in vitro* developed OLCs expressed some oocyte-specific genes [8, 9] and a variety of genes related to pluripotent stem cells [9], listed in Table 4. A big advantage of these cells is that they can be developed from autologous ovarian stem cells, even in women with severe ovarian infertility. 

Recently, Cheng et al. successfully developed stem cells from human amniotic fluid (hAFSCs) into OLCs [11]. After 15 days of cell culture and differentiation, OLCs with a diameter of 50–60 *μ*m and zona-pellucida- (ZP-) like structures were observed. They were trying to elucidate if the bone morphogenetic protein 15 (*BMP15*) gene was activated during the differentiation of human amniotic fluid stem cells into OLCs. When OLCs were analyzed by RT-PCR, *BMP15* was activated from approximately day 10 of cell differentiation. Additionally, the green-fluorescent-protein- (GFP-) BMP15 was transfected into the differentiating human amniotic stem cells, and its expression was positive in the OLCs. The RT-PCR analysis showed that the oocyte-specific genes, such as *ZP1*, *ZP2*, *ZP3*, and *c-kit,* were expressed in the differentiating hAFSCs, and the immunofluorescence assay confirmed that the ZP2 was detected in the OLCs. Using quantitative RT-PCR they found that that *ZP2* and *ZP3* expressions were significantly elevated in the differentiating stem cells. They concluded that the *BMP15* could be used as an important marker of oogenesis to follow the differentiation of amniotic fluid stem cells and other stem cells into the OLCs.

The results of all these studies showed that it is possible to trigger the development of OLCs from different types of stem cells by the addition of certain substances to the culture medium, such as bFGF, retinoic acid, forskolin or human/animal follicular fluid, or by using ovarian fibroblasts or mouse embryonic fibroblasts (MEFs) as a feeder layer (Table 4). The oocyte-like cells mostly had a diameter of around 60 *μ*m, and some of them expressed a zona-pellucida-like structure. In spite of some promising new knowledge, the existing results of genetic analyses showed that the OCLs developed *in vitro* are still far from real, fully competent human oocytes. They mostly failed to progress through the whole process of meiosis in spite of the complex *in vitro* maturation medium; therefore, the transplantation of these cells to mature them *in vivo* was proposed as a more reliable approach which may lead to potential clinical practice in the future [36]. Development of oocytes *in vitro* would be of great importance for the treatment of severe ovarian infertility (e.g., premature ovarian failure). Yet, in general, the OLCs which developed *in vitro* from human stem cells have not been genetically analyzed in greater detail, including the microarrays. This reveals the need to analyze the OLCs genetically and epigenetically in more detail in comparison with mature oocytes from the *in vitro* fertilization programme to evaluate their quality and safety in the future. At present the most important task is to better elucidate the expression of oocyte-specific genes (see Table 3). 

### 3.6. Gene Expression Profile and Oocyte *In Vivo* and *In Vitro* Maturation

The maturity of the human oocyte is one of the crucial features to sustaining a successful pregnancy to birth. It is known that immature human oocytes at the GV stage and MI stage of maturity cannot be fertilized. Human oocyte maturity is related to nuclear and cytoplasm maturity. Gasca et al. analyzed a pool of immature oocytes (20 GV oocytes in 7 patients and 20 MI oocytes in 7 patients), 37 non-fertilized mature MII oocytes, and cumulus cells by microarrays [2]. In all samples the gene expression level of *BRCA1* and *2*, *ATM*, *TP53*, *RB1*, *BUB1*, *MAD2*, *APC,* and *ACTB* was analyzed. The expression of *ACTB* and *MAD2* was present in all samples. Gene *RB1* was down-regulated in oocytes, while the genes *BUB1*, *BRCA1* and *2, *and *MAD2* were down-regulated in cumulus cells. The expression of *BRCA1 *and *2* was absent in cumulus cells, while the expression of *TP53* was absent in MI oocytes and *RB1* expression in MII oocytes. They identified new potential regulators and marker genes involved in the human *in vivo* oocyte maturation, such as *BARD1*, *RBL2*, *RBBP7*, *BUB3*, or *BUB1B *(Table 5). In the *RB1* group of transcription factors the expression of *RBBP8* was highest in the GV oocytes, *RBBP4* in MI oocytes, and *RBBP7* and *RBL2 *in MII oocytes. The high expression levels of *RBBP7*, *RBBP4,* and *RBL2* in MI and MII oocytes suggested that this transcription regulation pathway could be functional during oocyte maturation (Table 5). The expression of the gene *RBL1* was highly restricted for cumulus cells, indicating that this gene may be involved in the regulation of transcription in these cells. DNA repair markers *ATM* and *ATR* were differentially expressed during oocyte maturation; the expression of *ATR* mostly appeared in immature GV oocytes. Also the DNA repair marker *BARD1 *was expressed during oocyte maturation. The cell cycle checkpoint markers *BUB3* and *BUB1B* were expressed in immature MI oocytes, and the expression of gene *BRAP* was reduced in MII oocytes (see Table 5). Furthermore, Assou et al. [16] analyzed the total cRNA which was synthesized from pools of GV-, MI- or MII-stage oocytes, then labeled, and hybridized to pan-genomic oligonucleotide microarrays. Oocytes expressed on average 8,728 genes. The lowest number of genes was found to be expressed in MII oocytes (*n* = 5,633) and the highest number in GV oocytes (*n* = 10,892). On the other hand, in MII oocytes 444 genes were overexpressed, while in GV oocytes only 104 genes were overexpressed; in the transient MI oocytes only 4 genes were overexpressed. The expression variations between GV, MI, and MII oocytes were low. Similarly, Wells and Patrizio [26] found that GV oocytes expressed the highest number of genes, and *in vivo* matured MII oocytes the lowest number. Most of the differences between GV-stage oocytes and other stages were the consequence of a reduction in the number of mRNA transcripts occurring during progression to MII. This suggested that relatively little new gene expression occurred after the GV stage of maturity, with degradation of mRNA transcripts leading to the differences in transcript numbers observed at later stages of maturity in spite of the fact that the post-GV oocytes were not completely quiescent in terms of gene transcription, as revealed by the detection of some transcripts absent at the GV stage. Differently expressed genes were classified according to their molecular functions and biological processes. In Table 5 we can see the molecular function of most up-regulated genes in GV-stage oocytes in comparison to *in vivo *matured MII oocytes. For example, a number of genes for storage proteins displayed up-regulation in GV oocytes compared with *in vivo* matured MII oocytes.

*In vitro* maturation of human oocytes is an attractive strategy for *in vitro* fertilization treatment, especially in women with polycystic ovarian syndrome (PCOS) and with a risk of ovarian hyperstimulation after gonadotropin administration for oocyte retrieval. There is also a proportion of immature oocytes which are discarded in daily medical practice because of their immaturity. However, current *in vitro* maturation protocols unfortunately produce oocytes with a poor clinical outcome shown by poor embryo development and early pregnancy loss [37]. This is also reflected at the molecular level of *in vitro* matured oocytes. Wells and Patrizio analyzed *in vivo* matured oocytes at GV or MII stages of maturity and compared the gene expression profiles between MII oocytes matured *in vivo* and *in vitro* [26]. The non-fertilized oocytes from the *in vitro* fertilization programme were analyzed for more than 29,000 genes with RNA amplification and microarray technology. It was found that GV oocytes expressed 12,219 genes, *in vivo* matured MII oocytes 9,735, and *in vitro* matured MII oocytes 8,510. It is interesting that *in vivo* matured MII and *in vitro* matured MII oocytes shared very similar patterns of gene expressions, but they also noted some significant differences (see Table 6). It is interesting that some germ cell-specific genes (e.g., *DAZL*) and genes related to meiosis (e.g., *SYCP2*, *SGOL2*, and *MSH2*) were also among genes which were up-regulated in oocytes matured *in vitro*. In Table 6 we can see the molecular functions of genes which were expressed differently in oocytes matured *in vivo* versus *in vitro*. Some immature GV oocyte patterns of gene expression still persisted in MII oocytes matured *in vitro*. Although the *in vitro* matured MII oocytes closely resemble the *in vivo* matured oocytes, especially for genes related to nuclear maturity, there were several genes related to cytoplasmic functions which were expressed in an immature manner. It seems that the cytoplasmic maturation may be a crucial point regarding the worse clinical outcome obtained by *in vitro* matured MII oocytes. *In vivo* matured MII oocytes also expressed some genes associated with cellular storage and homeostasis differently. 

In the important further study by Jones et al. on global gene expression profiling using microarrays and bioinformatics a molecular basis for differences in the developmental competence of oocytes matured *in vitro* in comparison with *in vivo* matured oocytes was elucidated [27]. They found that more than 2,000 genes were identified as expressed at more than 2-fold higher levels in oocytes matured *in vitro* than those matured *in vivo*, and 162 of them were expressed at 10-fold or greater levels in oocytes matured *in vitro* (see Table 6). Many of these genes up-regulated in oocytes matured *in vitro* are involved in transcription, the cell cycle and its regulation, transport, and cellular protein metabolism. They came to an important conclusion that the overabundance of transcripts identified in immature GV-stage oocytes retrieved from gonadotropin stimulated cycles and matured *in vitro *in the programme of *in vitro* fertilization is probably due to deregulation in either gene transcription or posttranscriptional modification of genes. Each of the two proposed mechanisms would result in an incorrect temporal utilization of genes which may culminate in developmental incompetence of any embryos derived from these oocytes, as experienced in the clinical programme of *in vitro* fertilization. All these data indicated that the oocyte *in vitro* maturation procedure is far from optimal and needs to be further researched in the future. The relation between changed gene expression profile and the quality of oocytes is still poorly understood. Some attempts were made to relate the gene expressions to developmental competence of oocytes. O'Shea et al. [38] performed a meta-analysis on previously published microarray data on various models of oocyte and embryo quality and identified 56 candidate genes associated with oocyte quality across several species, including human. Moreover, they found that twenty-one potential biomarkers were associated with increased oocyte competence, and thirty-five potential biomarkers were associated with decreased oocyte competence (see Table 7). The up-regulation of METAP2 and the decrease of multiple genes linked to mRNA and protein synthesis in models of competence highlight the importance of *de novo* protein synthesis and its regulation for successful oocyte maturation and subsequent development. Also, the expression of ATRX and several other transcription factors were linked to decreased competence in oocytes (Table 7). These genes could potentially be used also as biomarkers of oocyte quality after the *in vitro* maturation procedure. The molecular genetic analyses of oocytes are an extremely important tool to provide a more efficient and safe procedure of oocyte *in vitro* maturation in the future. 

### 3.7. Gene Expression Profiles in Human Oocytes and *In Vitro* Fertilization Methods

Oocytes matured *in vitro* can be cryopreserved or fertilized in the *in vitro* fertilization programme. Cryopreservation is now considered to be an efficient way to store human oocytes to preserve fertility in young cancer patients before oncotherapy [39–41] (i.e., chemo- and radiotherapy) or in patients with no sperm on the day of *in vitro* fertilization [42]. There are two different principles in the preservation of human oocytes: stepwise—slow freezing and thawing in a liquid nitrogen vapour [43, 44]—and vitrification—the immediate plunge of oocytes into a vitrification solution with a high concentration of cryoprotectant and into liquid nitrogen [45–47]. There is a big debate over which method is of clinical preference. But in general, the clinical results of *in vitro* fertilization obtained by frozen-thawed MII oocytes are for the most part significantly worse than in fresh MII oocytes. In spite of the fact that these procedures are already in the clinical practice, the effects of these technologies on the oocyte gene expression are little known. Monzo et al. [28] studied the effect of these two different cryopreservation procedures, slow freezing and vitrification, on the gene expression profile of human MII oocytes. The gene expression profiles and associated biological pathways in slowly frozen/thawed and vitrified oocytes were compared with those of fresh control oocytes. It was found that both cryopreservation procedures negatively affected the gene expression profile of human oocytes in comparison with fresh controls. It is interesting that slowly frozen and vitrified oocytes displayed distinct gene expression profiles: slow oocyte freezing was associated with the down-regulation of genes related to chromosomal structure maintenance (e.g., *KIF2C* and *KIF3A*) and cell cycle regulation (e.g., *CHEK2* and *CDKN1B*) that may lead to a reduction in the oocyte developmental competence, while in vitrified oocytes many genes of the ubiquitination pathway were down-regulated, including those of the ubiquitin-specific peptidase family and subunits of the 26S proteasome. Such inhibition of the oocyte degradation machinery might stabilize the maternal protein content which is necessary for oocyte developmental competence. They concluded that the low pregnancy rates achieved by frozen-thawed human MII oocytes could be explained by the alterations of the oocyte gene expression profile. 

 Not only were the procedures of oocyte cryopreservation confirmed to be related to the changed oocyte gene expression profile, but so was the total fertilization failure (no fertilization of oocytes) in the *in vitro* fertilization programme. In patients with male factor infertility the total fertilization failure may be explained by the bad sperm quality, while in patients without male factor infertility the lack of identifiable criteria poses the question of the reliability of the clinical management. Gasca et al. analyzed the gene expression profiles of MII oocytes after total fertilization failure in a 30-year-old patient who had experienced three successive total fertilization failures (39 oocytes) and fertile control patients diagnosed with tubal or male infertility [29]. Transcriptional analysis of unfertilized MII oocytes revealed a total fertilization failure-altered gene expression profile with misexpression of genes related to meiosis, cell growth, and apoptosis control, all characterized by important fold changes. These results confirmed that even morphologically normal, high-grade oocytes in the *in vitro* fertilization programme may express some molecular abnormalities at the gene expression level, especially those related to the failure of MII oocyte activation. The authors proposed the microarray approach to improve the clinical therapeutic treatment and to offer informed counseling to the patients about alternate therapeutic solutions. These studies need to be performed with a higher number of oocytes to make a real conclusion.

### 3.8. Polar Body Gene Expression Profiling

In the *in vitro* fertilization programme some improved methods are needed to reliably evaluate oocyte quality prior to fertilization and to choose the right embryos to implant. Recent evaluation of oocyte quality is based only on the evaluation of morphology, but this is very limited, and also oocytes with high-grade morphology sometimes do not fertilize or develop into the embryo. mRNA and proteins produced during the oogenesis support embryonic development until the zygotic transition. Polar body gene expression profiling might be an important safe tool to verify the molecular status of *in vivo* or *in vitro* matured oocytes before the *in vitro* fertilization procedure. The first polar body is extruded from a mature oocyte before fertilization and can be biopsied without damaging the oocyte. The polar body transcriptome was proposed as a potential tool for the evaluation of oocyte quality in the *in vitro* fertilization programme [30, 31]. Klatsky et al. tested the hypothesis that mRNA originating from the expression in the meiotic MII oocyte from the *in vitro* fertilization programme is present and detectable in a single polar body prior to fertilization [30]. In their study immature oocytes from the intracytoplasmic sperm injection (ICSI) programme were cultured overnight and checked on the following day for their maturity. In all MII oocytes polar body biopsy was performed and followed by reverse transcription without RNA isolation. Sibling oocytes were prepared similarly to polar bodies. Then the complementary DNA from all samples was preamplified over 15 cycles for candidate genes using selective primers. Single-cell real-time PCR was performed to detect and quantify relative gene expression. It was important that polar body mRNA was detected for 11 of 12 candidate genes (*BCL2L10*, *ODC1*, *DPPA3*, *PADI6*, *DDX4*, *AGO2*, *DROSHA*, *GAPDH*, *EIF6*, *PABP*, *DICER*); interestingly, the oocyte-specific gene *H1FOO* was not expressed in any single polar body. Transcripts that were present in greater abundance in the single oocytes were also detected in qPCR replicates from single polar bodies. They concluded that preamplification of cDNA synthesized without RNA isolation can facilitate the quantitative detection of mRNA in single human polar bodies. This study was followed by the study by Reich et al. [31] who performed the first gene expression of microarray analyses of polar bodies. They performed a polar body biopsy on mature MII oocytes followed by the single-cell transcriptome analysis of the oocytes and their sibling polar bodies. They then compared over 12,700 mRNAs and miRNAs from the oocyte samples with the 5,431 mRNAs from the sibling polar bodies (5,256 shared mRNAs or 97%, including miRNAs). Their results showed that human polar bodies reflected the oocyte transcript profile. The valuable conclusion from their work is that mRNA detection and quantification through high-throughput quantitative PCR or microarrays could result in the first molecular diagnostics for gene expression profile of MII oocytes. The gene expression profiling of a polar body could enable the molecular diagnostics of oocyte quality in the programme of *in vitro* fertilization and might become a very important safe tool to evaluate the molecular status of *in vitro* matured oocytes and improve the clinical outcome of oocyte *in vitro* maturation in the future.

### 3.9. Oocyte Gene Expression Profile, Female Age, and Meiotic Aneuploidies

It is known that female fertility and development competence of human oocytes decline with increasing female age and that the proportion of oocytes with genetic abnormalities increased with female ageing. Steuerwald et al. found that the global gene expression profiles in human oocytes are related to female age [32]. Their results confirmed that the expression of oocyte genes related to major functional processes and features, including cell cycle regulation, cytoskeletal structure, energy pathways, transcription control, and stress responses depends on female age. Similarly, Grøndahl et al. analyzed the single mature MII non-fertilized oocytes from two groups of patients—younger (<36 years) and older (37–39 years)—in the *in vitro* fertilization programme [33]. Based on 15 independent replicates of single MII oocytes, 7,470 genes (10,428 transcripts) were identified in the MII oocytes. Among these genes, 342 were expressed at a significantly different expression level between the two age groups of patients. These genes were found to be involved in cell cycle regulation, chromosome alignment (e.g., MAD2L1 binding protein), sister chromatid separation (e.g., separase), oxidative stress, and ubiquitination. The top signaling network of genes, which was proven to be affected by female age, was “cell cycle and organism development” (e.g., *SMAD2* and activin B1 receptor). It was concluded that these genes may be associated with the ageing process and decreased fertility. Advanced female age may be also related to oocyte aneuploidy (the abnormal number of chromosomes). Fragouli et al. confirmed the link between mRNA transcript numbers in oocytes and female age [34]. They combined a comprehensive cytogenetic investigation of 21 oocytes with a detailed assessment of their transcriptome. The first polar body was removed from each oocyte and aneuploidy assessed using comparative genomic hybridization. Then the mRNA transcript data were produced using microarrays for seven oocytes, three normal and four aneuploid. The results showed that 327 genes were differently expressed in both groups of oocytes at statistical significance, and they provided the list of these genes. Ninety-six of these genes were further assessed by RT-PCR. The results confirmed that oocyte aneuploidy was associated with altered transcript levels affecting a subset of genes. They concluded that different transcript levels in normal and aneuploid oocytes may have an impact on cellular pathways, such as meiotic spindle assembly, chromosome alignment, production of cell surface or excretory molecules, and might potentially serve as targets for noninvasive oocyte aneuploidy assessment. This may explain the decline of female fertility and increase in spontaneous abortion with age. 

When talking about the oocyte *in vitro* maturation procedure, it is more appropriate to be applied in younger women because of a higher risk of oocyte genetic abnormalities in older women. Increased female age may be an important contraindication for the oocyte *in vitro* maturation procedure. 

All these new findings provide some new knowledge and better insights into human oocyte quality and oogenesis that might be introduced in clinical practice and might also lead to successful *in vitro* oogenesis in women with no naturally present oocytes in the future. 

## Figures and Tables

**Table 1:** Analyses of human oocyte gene expression by RT-PCR.

Oocyte gene expression analyses by RT-PCR				
Analyzed oocytes	Maturity of oocytes	Expression of genes	Functions of analyzed genes	Study
Four oocytes, forty-two embryos	MII, non-fertilized	High expression of *BRCA1*, *BRCA2*, *ATM*, *TP53*, *RB1*, *MNAD2*, *BUB1*, *APC*, *beta-actin* in oocytes, but low in embryos	Genes related to the preimplantation development	Wells et al., 2005 [17]

Four single oocytes, five embryos	MII, non-fertilized	*LDH-A*, *LDH-B*, *LDH-C* expression in oocytes and embryos	Genes encoding lactate dehydrogenase isozymes	
		*SUMO-1*, *SUMO-2*, *SUMO-3* expression in oocytes and embryos	Small ubiquitin-like modifier isoforms	Li et al., 2006 [18]

Oocytes from follicles	From primordial follicles to MII oocytes	*NOBOX* expression from primordial follicles to MII oocytes	Homeobox genes encoding transcription factors	
	From primordial to early primary follicles	*HOXA10* expression from primordial to early primary follicles		
	From primordial follicles to GV oocytes	*HOXB7* expression from primordial follicles to GV oocytes		
	GV oocytes	*HOXA7* expression in GV oocytes		Huntriss et al., 2006 [19]
	MII oocytes	*HOXA1*, *HEX* expression in MII oocytes		
	Cumulus cells	*HOXC9*, *HOXC8*, *HOXC6*, *HOXA7*, *HOXA5*, *HOXA4* expression in cumulus cells		

**Table 2:** Gene expression profiling of human oocytes using microarrays.

Analyses of oocyte gene expression by microarrays			
Analyzed oocytes	Expression of genes	Functions of analyzed genes	Study
Seventy seven *in vivo* matured oocytes at different stages of maturity: 20 GV oocytes (7 patients), 20 MI oocytes (7 patients), 37 MII oocytes, cumulus cells	Identification of new potential regulators and marker genes involved in the human *in vivo* oocyte maturation	Transcription regulation, DNA repair, cell cycle checkpoint	Gasca et al., 2007 [2]

Individual MII oocytes and groups of 5 MII oocytes	1,361 transcripts expressed in oocytes	Apoptosis, cell cycle, circadian rhythms, cytoskeleton, secretory pathways, exocytosis, endocytosis, kinases, membrane receptors, ion channels, mitochondria, structural nuclear proteins, phospholipases, protein degradation and synthesis, secreted proteins, signaling pathways, DNA, chromatin, RNA, transcription, and others	Bermúdez et al., 2004 [14]

Groups of 10 MII oocytes from women aged <35 years in comparison to 10 different normal human somatic tissues	5,331 transcripts significantly up-regulated and 7,074 transcripts significantly down-regulated in human oocytes	Up-regulated TGF-*β* pathway, DNA, RNA and protein metabolism, transcription regulation, chromatin modification	Kocabas et al., 2006 [15]

Groups of 20 GV, 20 MI, and 16 MII oocytes	Oocytes expressed in average 8,728 genes. The lowest number of expressed genes in MII oocytes (5,633) and highest in GV oocytes (10,892)	Genes specifically expressed in germinal cells and oocytes, meiosis, components of the maturation-promoting factor (MPF), spindle checkpoint, transforming growth factor-beta superfamily, chromatin remodeling	Assou et al., 2006 [16]

Nine MII and GV oocytes, preimplantation embryos	Human oocytes are low RNA template samples and an amplification step is required to provide sufficient labeled RNA as a microarray target (PCR and serial analysis of gene expression SAGE, microarrays)		Neilson et al., 2000 [20] Dobson et al., 2004 [21]

Seven individuals: 5 GV (primary) and 2 MII (secondary) oocytes and 15 preimplantation embryos	Down-regulation of genes in preimplantation embryos in comparison with oocytes	Oocyte maturation and embryo development	Dobson et al., 2004 [21]

Single and pooled GV oocytes	Oocytes need to be pooled for the starting template for each array and sufficient microarray experiments performed to minimize the variance associated with processing		Jones et al., 2007 [22]

Four individual MII oocytes and four 4-cell and three 8-cell embryos	A total of 631 genes exhibited differential expression in oocytes and embryos. In oocytes 184 genes were expressed more than twofold above the median value. Only two genes were at least twofold below the median value	Interconversion of lactate and pyruvate, lactate dehydrogenase, oocyte maturation, embryo development	Li et al., 2006 [18]

Immature oocytes from primordial, intermediate, and primary follicles	A total of 6,301 unique genes were significantly expressed; extraordinary high expression levels of *TMEFF2*, *OPHN1* and *ATP6*; expression of oocyte- or germline-specific genes	RNA binding, translation initiation structural molecule activity, BMP receptors, activin receptors, IGFI receptor, fibroblast growth factors, different enzymes	Markholt et al., 2012 [23]

Seventy six GV oocytes from 55 donor patients, hESCs, and human foreskin fibroblasts	10,183 genes were expressed in GV oocytes including oocyte-specific genes. Distinct sets of genes were detected in oocytes, hESCs and fibroblasts	In GV oocytes 4 signaling pathways—MOS-MPF, transforming growth factor-beta, Wnt, and Notch, oocyte maturity, embryo development	Zhang et al., 2007 [24]

GV and MII oocytes, hESCs, somatic tissues	Identified a common oocyte/hESC gene expression profile	Cell cycle, enzymes involved in general cell metabolism, nucleoside synthesis, DNA repair, cell cycle regulatory machinery, regulation of the topologic state of DNA, mitotic spindle assembly checkpoint, pluripotency, chromatin remodelling, transcription factors, ubiquitination, and proteasome pathways	Assou et al., 2009 [25]

GV oocytes, MII oocytes matured *in vivo* and *in vitro *	GV, *in vivo* matured MII oocytes, and *in vitro* matured MII oocytes expressed 12,219, 9,735, and 8,510 genes. There was an extensive overlap among the all three groups of oocytes, but also some significant differences. There were some immature GV oocyte patterns of gene expression, which still persisted in *in vitro* matured oocytes	Nuclear maturity, cytoplasmic functions expressed in an immature manner, cellular storage and homeostasis	Wells and Patrizio, 2008 [26]

GV oocytes, MII oocytes matured *in vivo* and *in vitro *	More than 2,000 genes were expressed at more than 2-fold higher levels in oocytes matured *in vitro* than those matured *in vivo *	Transcription, the cell cycle and its regulation, transport and cellular protein metabolism	Jones et al., 2008 [27]

Fresh, slowly frozen, and vitrified MII oocytes	Oocyte slow freezing and vitrification negatively affected the gene expression profile of human oocytes in comparison with fresh controls	Chromosomal structure maintenance, cell cycle regulation, genes of the ubiquitination pathway	Monzo et al., 2012 [28]

Thirty nine MII oocytes with total fertilization failure and control oocytes	Misexpression of several genes, characterized by important fold changes in oocytes with total fertilization failure	Meiosis, cell growth, and apoptosis control	Gasca et al., 2008 [29]

Fifteen GV oocytes which matured to MII stage overnight and their polar bodies	Transcripts that were present in greater abundance in the single oocytes were also detected in qPCR replicates from single polar bodies, except oocyte-specific *H1FOO *		Klatsky et al., 2010 [30]

Single MII oocytes and single polar bodies after biopsy	Human polar bodies reflected the oocyte transcript profile. 5,256 mRNAs, or 97%, including miRNAs were expressed in both oocytes and polar bodies		Reich et al., 2011 [31]

MII oocytes of younger (<32 years) and older women (>40 years)	Found that the global gene expression profiles in oocytes are related to female age. Genes were down-regulated in older women	Cell cycle regulation, cytoskeletal structure, energy pathways, transcription control, and stress responses	Steuerwald et al., 2007 [32]

Single MII oocytes of younger (<34 years) and older women (37–39 years)	7,470 genes (10,428 transcripts) were expressed in oocytes; 342 genes were expressed at significantly different expression levels between the two age groups of patients	Cell cycle regulation, chromosome alignment, sister chromatid separation, oxidative stress and ubiquitination, the signaling network of genes for cell cycle and organism development	Grøndahl et al., 2010 [33]

Seven MII oocytes (three normal and four aneuploid) and their polar bodies after biopsy	At comparative genomic hybridization 327 genes were differently expressed in both groups of oocytes; the relation between mRNA transcript numbers and female age	Meiotic spindle assembly, chromosome alignment, production of cell surface, or excretory molecules.	Fragouli et al., 2010 [34]

**Table 3:** Some of genes significantly overexpressed in oocytes according to [15, 16] and GeneCards data.

Genes significantly overexpressed in oocytes		
Gene symbol	Gene title	Chromosome
Gamete markers		
*DAZL *	Deleted in azoospermia-like	3
*DDX4/VASA *	DEAD (Asp-Glu-Ala-Asp) box polypeptide 4	5
*DPPA3/STELLA *	Developmental pluripotency associated 3	12

Embryogenesis, pluripotency, self-renewal, proliferation, development		
*ZAR1 *	Zygote arrest 1	4
*PUM1 *	Pumilio homolog 1 (*Drosophila*)	1
*PUM2 *	Pumilio homolog 2 (*Drosophila*)	2
*NANOS1 *	Nanos homolog 1 (*Drosophila*)	10
*NANOG *	Nanog homeobox	12
*SOX2 *	SRY (sex determining region Y)-box 2	3
*SALL2 *	Sal-like 2 (*Drosophila*)	14
*KLF4 *	Kruppel-like factor 4 (gut)	9
*LIN28B *	Lin-28 homolog B (*C. elegans*)	6

Maturation promoting and related factors		
*CCNB1 *	Cyclin B1	5
*CCNB2 *	Cyclin B2	15
*CDC2/CDK1 *	Cell division cycle 2, G1 to S and G2 to M	10
*CDC25A *	Cell division cycle 25A	3
*CDC25B *	Cell division cycle 25B	20
*CDC25C *	Cell division cycle 25C	5

Spindle checkpoint		
*BUB1 *	BUB1 budding uninhibited by benzimidazoles 1 homolog	2
*BUB1B/BUBR1 *	BUB1 budding uninhibited by benzimidazoles 1 homolog beta	15
*CENPA *	Centromere protein A	2
*CENPE *	Centromere protein E	4
*CENPH *	Centromere protein H	5
*MAD2L1/MAD2 *	MAD2 mitotic arrest deficient-like 1	4

Cytoplasmic receptors		
*NALP5/NLRP5/MATER *	NLR family, pyrin domain containing 5	19

APC/C complex, securin, cohesins		
*ANAPC1/APC1 *	Anaphase promoting complex subunit 1	2
*ANAPC10/APC10 *	Anaphase promoting complex subunit 10	4
*CDC20 *	CDC20 cell division cycle 20	1
*PTTG1 *	Pituitary tumor-transforming 1	5
*PTTG3 *	Pituitary tumor-transforming 3 (meiosis)	8
*STAG3 *	Stromal antigen 3 (meiosis)	7

Epigenetic remodeling		
*DNMT1 *	DMA (cytosine-5-)-methyltransferase 1	19
*DNMT3B *	DMA (cytosine-5-)-methyltransferase 3 beta	20
*HDAC9 *	Histone deacetylase 9	7
*H1FOO *	H1 histone family, member O, oocyte-specific	3
*HCAP-G *	Chromosome condensation protein G	4

Meiosis, miscellaneous		
*AKAP1 *	A kinase (PRKA) anchor protein 1	17
*MCM3 *	MCM3 minichromosome maintenance deficient 3	6
*MOS *	v-mos Moloney murine sarcoma viral oncogene homolog	8
*REC8 *	REC8 homolog (yeast)	14
*STAG3 *	Stromal antigen 3	7
*FMN2 *	Formin 2	1
*SYCP1 *	Synaptonemal complex protein 1	1
*SYCP2 *	Synaptonemal complex protein 2	20
*SYCP3 *	Synaptonemal complex protein 3	12
*SMC3 *	Structural maintenance of chromosomes 3	10
*SMC1B *	Structural maintenance of chromosomes 1B	22
*STRA8 *	Stimulated by retinoic acid gene 8 homolog (mouse)	7
*MLH1 *	mutL homolog 1, colon cancer, nonpolyposis type 2 (*E. coli*)	3
*DMC1 *	DMC1 dosage suppressor of mck1 homolog, meiosis-specific homologous recombination (yeast)	22
*PELO *	Pelota homolog (*Drosophila*)	5
*WEE2 *	WEE1 homolog 2 (*S. pombe*)	7
*SGOL2 *	Shugoshin-like 2 (*S. pombe*)	2
*PPP2CA *	Protein phosphatase 2, catalytic subunit, alpha isozyme	5
*SPAG16 *	Sperm associated antigen 16	2
*TUBB4Q *	Tubulin, beta polypeptide 4, member Q	4
*FBXO5/EMI1 *	F-box protein 5	6
*AURKC *	Aurora kinase C	19

Extracellular matrix, growth factors, cell surface, signaling		
*BMP15 *	Bone morphogenetic protein 15	X
*BMP6 *	Bone morphogenetic protein 6	6
*GDF9 *	Growth differentiation factor 9	5
*FGFR2 *	Fibroblast growth factor receptor 2	10
*FGF9 *	Fibroblast growth factor 9 (glia-activating factor)	13
*FGF14 *	Fibroblast growth factor 14	13
*KIT *	v-kit Hardy-Zuckerman 4 feline sarcoma viral oncogene homolog	4
*IL4 *	Interleukin 4	5
*TNFSF13/APRIL *	Tumor necrosis factor superfamily, member 13 v-erb-a erythroblastic leukemia viral oncogene	17
*ERBB4 *	Homolog 4	2
*FZD3 *	Frizzled homolog 3	8
*GPR37 *	G protein-coupled receptor 37 (endothelin receptor type B-like)	7
*GPR39 *	G protein-coupled receptor 39	2
*GPR51 *	G protein-coupled receptor 51	9
*GPR126 *	G protein-coupled receptor 126	6
*GPR143 *	G protein-coupled receptor 143	X
*GPR160 *	G protein-coupled receptor 160	3
*ZP1 *	Zona pellucida glycoprotein 1 (sperm receptor)	11
*ZP2 *	Zona pellucida glycoprotein 2 (sperm receptor)	16
*ZP3 *	Zona pellucida glycoprotein 3 (sperm receptor)	7
*ZP4 *	Zona pellucida glycoprotein 4	1
*SLC5A11 *	Solute carrier family 5 (sodium/glucose cotransporter), member 11	16
*SOCS7 *	Suppressor of cytokine signaling 7	17

Transcription factors, oogenesis, folliculogenesis		
*FIGLA *	Folliculogenesis specific basic helix-loop-helix	2
*POU5F1 *	POU class 5 homeobox 1	6
*NOBOX *	NOBOX oogenesis homeobox	7
*BNC1 *	Basonuclin 1	15
*GCNF/NR6A1 *	Nuclear receptor subfamily 6, group A, member 1	9
*SOX15 *	SRY (sex determining region Y)-box 15	17
*SOX30 *	SRY (sex determining region Y)-box 30	5
*OTX2 *	Orthodenticle homolog 2 (*Drosophila*)	14
*FOXR1 *	Forkhead box R1	11
*JARID2 *	Jumonji, AT rich interactive domain 2	6

Chromatin reprogramming		
*NPM2 *	Nucleophosmin/nucleoplasmin 2	8

Postreplicative DNA mismatch repair system		
*MSH2 *	mutS homolog 2, colon cancer, nonpolyposis type 1 (*E. coli*)	2

Oolema receptor (oocyte-sperm adhesion)		
*ASTL *	Astacin-like metalloendopeptidase (M12 family)	2

Imprinted genes		
*MEST *	Mesoderm specific transcript homolog	7

Apoptosis		
*BNIP1 *	BCL2/adenovirus E1B 19 kDa interacting protein 1	5
*BIRC5 *	Baculoviral IAP repeat-containing 5 (survivin)	17
*BCL2L10 *	BCL2-like 10 (apoptosis facilitator)	15

**Table 4:** Expression of genes in oocyte-like cells developed *in vitro* from human stem cells of different sources.

Gene expression of oocyte-like cells developed *in vitro* from stem cells in humans			
Source	Critical culture condition	Expression of genes (method of detection)	References
Human embryonic stem cells (hESCs)	bFGF, feeder layer (mouse embryonic fibroblasts)	Premigratory/migratory genes related to pluripotency: *IFITM3*, *DAZL*, *NANOG*, *POU5F1*, postmigratory genes: *PIWIL2*, *PUM2* and genes related to meiosis: *SYCP3* and *MLH1 *(RT-PCR).	West et al., 2008 [3]

hESCs	bFGF, feeder layer (mouse embryonic fibroblasts)	Germ cell-related gene *DDX4* (*VASA*) and pluripotency-related *POU5F1 *(*OCT4*). (RT-PCR)	West et al., 2010 [4]

hESCs	Ovarian fibroblasts	*VASA*,* DAZL*,* GDF3*,* GDF9*,* MLH1*, *SCP1*,* PUM1*, *PUM2*,* POU5F1 *(RT-PCR)	Richards et al., 2010 [5]

hESCs Human-induced pluripotent stem cells (hiPSCs)	Culture medium with glutamine, 2-mercaptoethanol and bFGF	Early germ cell markers:*VASA*, *DAZL*,* IFITM1*,* PRDM1A*,* GCNF*,* GDF3*,* CKIT*,* PELOTA* Late germ cell and meiotic markers:* SCP3*,* MLH1*,* DMC1*,* GDF9*, *ZP4* (Biomark 96.96 microfluidic qPCR chip Fluidigm).	Medrano et al., 2012 [6]

hESCs hiPSCs from keratinocytes and umbilical cord blood	Culture medium with retinoic acid, forskolin, and bFGF	*OCT4*, *NANOG*, *SOX2*, *KLF4*, *C-MYC*, *DPPA4*, *DNMT3B*, *REX1*, *SALL2*, *LIN28, VASA,*and* STRA8 *(RT-PCR) Haploid cells confirmed by fluorescence in situ hybridization for chromosomes X, Y, and 18	Eguizabal et al., 2011 [7]

Stem cells from adult ovarian surface epithelium (hOSCs)	Culture medium with phenol red (weak estrogenic stimuli)	*OCT4A*, *OCT4B*, *c-KIT*, *VASA*, and *ZP2 *(RT-PCR)	Virant-Klun et al., 2008 [8]

Stem cells from adult ovarian surface epithelium (hOSCs)	Culture medium with added follicular fluid	*OCT4A*, *SOX-2*, *NANOG*, *NANOS*, *STELLA*, *CD9*, *LIN28*, *KLF4*, *GDF3*, and *MYC *(Single-Cell Gene Expression Analyses by Fluidigm BioMark System)	Virant-Klun et al., 2011 [10]

Amniotic fluid stem cells (hAFSCs)	Culture medium with glutamine, beta-mercaptoethanol, and porcine follicular fluid	*BMP15*, *ZP1*, *ZP2*, *ZP3*, *c-kit, VASA* (RT-PCR) BMP15 was proposed as an important marker of oogenesis	Cheng et al., 2012 [11]

**Table 5:** Genes which were significantly up-regulated in human oocytes at different stages of maturity.

Up-regulated genes at different stages of oocyte maturity			
GV (immature)	MI (immature)	MII (mature)	References
*RBBP8*, *ATR *	*BRAP*, *BUB1B*, *BUB3 *	*RBBP7*, *RBL2 *	Gasca et al., 2007 [2]
*GBX1*, *HOXA7 *		*HOXA1*, *HEX *	Huntriss et al., 2006 [19]

Molecular functions of differently expressed genes			
(GV versus MII oocytes)			
GV Up-regulated genes (number)		GV Down-regulated genes (number)	References

(i) Nucleic acid binding (149)		(i) Nucleic acid binding (235)	
(ii) Zinc finger transcription factor (58)		(ii) Ribosomal protein (101)	
(iii) KRAB box transcription factor (41)		(iii) Receptor (39)	
(iv) Ligase (31)		(iv) Translation factor (22)	
(v) Chaperone (25)		(v) Ribonucleoprotein (15)	Wells and Patrizio, 2008 [26]
(vi) Synthase and synthetase (25)		(vi) Translation elongation factor (14)	
(vii) Receptor (22)		(vii) Signaling molecule (13)	
(viii) Other RNA-binding factors (19)		(viii) G-protein coupled receptor	
(ix) mRNA processing factor (17)		(ix) Tubulin	

**Table 6:** Genes which were differently expressed in human oocytes matured *in vitro* in comparison with oocytes matured *in vivo* at a cut-off value of 10-fold or higher [26, 27].

Genes differently expressed in oocytes matured	
*in vitro* or *in vivo* [27]	
Significantly up-regulated genes in oocytes matured *in vitro *	Significantly down-regulated genes in oocytes matured *in vitro *
*HNRNPA2B1*,* TCEB1*, *HINT1*,* PNPLA1*,* PAOX, NLRP12*,* KIF23*,* PRPF38B*,* UBA52*, *GEM*, *MBD4*, *MORF4L2*,* ATF2*,* AFF4*,* ZCCHC14*,* ZNF669*,* CCNG2*,* NFE2L2*, MSH2 *, *ZNF610*, *C21orf66*,* CDC37L1*,* UPF3B*,* TAOK3*, *GPR64*,* HSPA14*,* C18orf24*,* TAF1A*,* MAP2K7*,* ZNF571*,* PFKFB2*,* CUL1*,* CLDN10*,* CLK1*, *RCOR1*, *ZPBP2*,* GTF2H2*,* TSG101*,* ARHGAP11A*,* HPS3*,* GRHL1*,* EIF4G2*, *RFC4*, *VPS26A*,* PIK3C2A*,* GDI2*,* RAB23*, *WDSOF1*,* PLCL1*,* DCDC2*,* MINPP1*, *PTPN12*,* METAP2*,* LHX8*,* LOC91664*, SYCP2 *,* ATP5L*,* ICK*,* FUCA1*,* SMARCAD1*,* BRD7*, *TBPL1*,* ABCD3*,* SSR3*,* FEZ2*,* FHOD3*,* AMD1*, DAZL *,* ZMYM2*,* FANCL*,* SLCO1A2*,* NUFIP1*,* STAU2*,* PCF11*,* PPP1R3C*,* POLI*, SGOL2 *,* PBLD*, *CCNL1*,* SLBP*,* MRPS30*, *TMOD2*,* FBXW11*,* CDK7*,* HNRNPR*, *CFDP1*,* DEPDC7*, *CTSL2*, *DYNC1I2*,* NCKAP1*, *TRPA1*,* DCLRE1A*,* RAB18*,* GTDC1*,* LOC283514*, *CLUL1*,* ZNF131*,* SP3*,* ZNF302*,* MAD2L1*, *TRAPPC6B*,* C10orf137*,* MTPN*, *ZNF313*,* PLEKHA3*,* ZCCHC14*,* RTTN*,* ZNF136*,* ADH5*,* CCDC88A*,* CPEB2*,* SPATA5L1*,* UGP2*,* UGP2*,*MFSD1*,* TMEM27*,* ZNF443*,* UAP1*,* MIB1*,* MUT *	*HTRA1, CCDC69, FHL2 *

Molecular functions of differently expressed genes	
(*in vitro* versus *in vivo* matured oocytes) [26]	
Significantly up-regulated genes in oocytes matured *in vitro* (number)	Significantly down-regulated genes in oocytes matured *in vitro* (number)

(i) Nucleic acid binding (62)	(i) Nucleic acid binding (75)
(ii) Zinc finger transcription factor (13)	(ii) Ribosomal protein (37)
(iii) Other RNA-binding factors (10)	(iii) Translation factor (8)
(iv) Ligase (9)	(iv) Translation elongation factor (6)
(v) KRAB box transcription factor (8)	(v) Storage protein (6)
(vi) mRNA processing factor (8)	(vi) Receptor (5)
(vii) Chaperone (7)	(vii) Tubulin (4)
(viii) Receptor (7)	(viii) Signaling molecule (3)
(ix) Signaling (7)	(ix) Ribonucleoprotein (2)
(x) Ribonucleoprotein (7)	(x) Defense/immunity protein (1)

*Genes related to germ cells and meiosis.

**Table 7:** Transcription factors associated with developmental competence of oocytes found by meta-analysis of previously published microarray studies comparing *in vivo* and *in vitro* matured oocytes in bovine and monkey, human healthy and polycystic ovaries (PCOS-polycystic ovary syndrome), and mouse young and aged ovaries [38].

Transcripts associated with developmental competence of oocytes			
Increased developmental competence		Decreased developmental competence	
CPD	Carboxypeptidase D precursor	PRKG1	cGMP-dependent protein kinase 1, alpha isozyme
SH3BGRL	SH3 domain binding glutamic acid-rich-like protein	IGFBP3	Insulin-like growth factor binding protein 3
CDC123	Cell division cycle 123	DUSP1	Dual specificity phosphatase 1
AQP1	Aquaporin 1	NDRG4	Ndr4
GNB5	Guanine nucleotide binding protein beta 5	ATOX1	Copper transport protein
METAP2	Methionine aminopeptidase 2	TMSB10	Thymosin beta-10
UBE2E3	Ubiquitin-conjugating enzyme E2E3	ATRX	Alpha thalassemia/mental retardation syndrome X-linked
USP6NL	USP6 N-terminal-like protein	SHMT2	Serine hydroxymethyltransferase 2
HMGA1*	**High Mobility group AT-hook1**	TGFBR3*	**Transforming growth factor beta receptor III**
TCTEL1	TCTEL1 protein	FANK1	Fibronectin type 3 and ankyrin repeat domains 1 protein
SNRPD3	Small nuclear ribonucleoprotein D3 polypeptide	PTPN1	Protein tyrosine phosphatase, nonreceptor type 1
MTG1	Mitochondrial GTPase 1 homolog	DDX55	ATP-dependent RNA helicase DDX55
GKAP1	G kinase anchoring protein 1	SFRP1*	**Secreted frizzled-related protein 1 **
UFM1	Ubiquitin-fold modifier 1	BMP4	Bone morphogenetic protein 4
MITD1	Mitochondria interacting transport domain 1	HIST1HBG	Histone H2B type 1-C/E/G
MRPL3	Mitochondrial ribosomal protein L3	SEC61G	Protein transport protein Sec61 subunit gamma
KIF23	Kinesin family member 23	FOXM1*	**Forkhead box protein M1 **
DDX52	DEAD box polypeptide 52	NDUFA1	NADH dehydrogenase 1 alpha subcomplex subunit 1
		TUSC4	Tumor suppressor candidate 4
		JMJD1C	Jumonji domain containing 1C
		POL2RL	DNA-directed RNA polymerases I, II, and III subunit RPABC5
		MED1	Mediator complex subunit 1
		SLC39A14	Solute carrier family 39 member 14
		CALM2	Calmodulin 2
		RPS18	40S ribosomal protein S18
		INVS	Inversion
		RBMS1	RNA binding motif single stranded interacting protein 1
		PLXNC1	Plexin C1
		BTF3l4	Basic transcription factor 3-like 4
		MAP3K12*	**Mitogen-activated protein 3 kinase 12**
		PXMP4	Peroxisomal membrane protein 4
		EG216818	Ubiquitin
		JAM2	Junctional adhesion molecule 2
		IFITM1	Interferon-induced transmembrane protein 1
		LRRC28	Leucine rich repeat containing 28

Transcription regulation			
		Associated with decreased competence in oocytes	

		E2f4 65	
		Sp3 61	
		Gata-1 62	
		C/ebp *β* 62	
		Rela (P65 NF-Kb Subunit)	

*Potential biomarkers of oocyte developmental competence.
